# Multiple Roles of Ret Signalling During Enteric Neurogenesis

**DOI:** 10.3389/fnmol.2022.832317

**Published:** 2022-05-27

**Authors:** Dipa Natarajan, Conor McCann, Justine Dattani, Vassilis Pachnis, Nikhil Thapar

**Affiliations:** ^1^Division of Molecular Neurobiology, MRC National Institute for Medical Research, London, United Kingdom; ^2^Birth Defects Research Centre, Great Ormond Street Institute of Child Health, University College London, London, United Kingdom; ^3^Department of Mathematical Sciences, University of Bath, Bath, United Kingdom; ^4^The Francis Crick Institute, London, United Kingdom; ^5^Department of Gastroenterology, Hepatology and Liver Transplant, Queensland Children’s Hospital, Brisbane, QLD, Australia; ^6^Faculty of Medicine, University of Queensland, Brisbane, QLD, Australia; ^7^Woolworths Centre for Child Nutrition Research, Queensland University of Technology, Brisbane, QLD, Australia

**Keywords:** enteric nervous system, neural crest cells, Hirschsprung disease, colonic aganglionosis, normoganglionic gut, ENS progenitor cells, isoforms

## Abstract

The majority of the enteric nervous system is formed by vagal neural crest cells which enter the foregut and migrate rostrocaudally to colonise the entire length of the gastrointestinal tract. Absence of enteric ganglia from the distal colon are the hallmark of Hirschsprung disease, a congenital disorder characterised by severe intestinal dysmotility. Mutations in the receptor tyrosine kinase RET have been identified in approximately 50% of familial cases of Hirschsprung disease but the cellular processes misregulated in this condition remain unclear. By lineage tracing neural crest cells in mice homozygous for a knock-in allele of *Ret* (*Ret^51/51^)*, we demonstrate that normal activity of this receptor is required *in vivo* for the migration of enteric nervous system progenitors throughout the gut. In mutant mice, progenitors of enteric neurons fail to colonise the distal colon, indicating that failure of colonisation of the distal intestine is a major contributing factor for the pathogenesis of Hirschsprung disease. Enteric nervous system progenitors in the ganglionic proximal guts of mutant mice are also characterised by reduced proliferation and differentiation. These findings suggest that the functional abnormalities in Hirschsprung disease result from a combination of colonic aganglionosis and deficits in neuronal circuitry of more proximal gut segments. The reduced neurogenesis in the gut of *Ret^51/51^* mutants was reproduced in the multilineage enteric nervous system progenitors isolated from these animals. Correction of the molecular defects of such progenitors fully restored their neurogenic potential in culture. These observations enhance our understanding of the pathogenesis of Hirschsprung disease and highlight potential approaches for its treatment.

## Highlights

- Normal *Ret* activity is required for migration, proliferation and neuronal differentiation of neural crest cells (NCC).
- The *Ret*^51/51^ mouse line (homozygous for a knock-in mutation expressing only the Ret^51^ isoform) is an established animal model of human Hirschsprung Disease.
- *Ret*^51^/YFP line generated to lineage trace NCC in these animals.
- Neuronal circuitry is affected in the proximal “normoganglionic” region of *Ret*^51/51^ gut as shown by DiI labelling of the nerve plexuses.
- ENS progenitor cells (EPCs) derived from the ganglionic segment of *Ret*^51/51^ guts exhibit defects *in vitro.*
- *Ret*^51/51^ EPCs can be rescued by introducing the *Ret*^9^ isoform.
- Proximal segments of *Ret*^51/51^ gut may phenocopy the dysfunction commonly observed in the proximal ganglionated bowel in Hirschsprung Disease.
- Study enhances our understanding of Hirschsprung disease and highlights potential treatment approaches.

## Introduction

The enteric nervous system (ENS) is composed of a large number of neurons and glia which form interconnected ganglia that control the peristalsis, blood flow and secretions of the gut wall (Furness, 2006). In mice, most progenitors of the ENS originate at embryonic day (E) 8.5–8.75 from the vagal neural crest (NC) and migrate ventrolaterally to reach the dorsal aorta. These pre-enteric NC cells (pENCCs) invade the foregut mesenchyme and (thereafter called enteric NC cells-ENCCs) initiate their rostrocaudal migration to colonise, uniformly, the entire length of the gastrointestinal tract (Kapur et al., 1992; Lo and Anderson, 1995; Durbec P. et al., 1996; Durbec P. L. et al., 1996; Pattyn et al., 1999; Young et al., 2001; Burns, 2005). During migration, ENCCs receive signals that allow them to survive, proliferate extensively and differentiate into enteric neurons and glia (Heanue and Pachnis, 2007; Laranjeira et al., 2011; Sasselli et al., 2012; Akbareian et al., 2013).

Hirschsprung disease (HSCR) is a congenital condition characterized by a failure of the ENS to complete development along the length of the gastrointestinal (GI) tract, which results in the absence of enteric ganglia in the most distal segment of the large intestine, variably extending more proximally. This leads to tonic muscle contraction of the affected part resulting in functional intestinal obstruction and, if left untreated, toxic megacolon and death (Amiel et al., 2008). Surgical resection of the aganglionic gut segment remains the treatment of choice for HSCR patients as it alleviates the life-threatening consequences of obstruction (Haricharan and Georgeson, 2008). However, a significant proportion, if not the majority, of HSCR patients are characterised post-operatively by high levels of morbidity (Metzger et al., 2009) although it is currently unclear whether this results from the primary pathology (aganglionosis) and the necessary surgical intervention or from additional deficits in the maturation and function of neuronal networks in more proximal and apparently “normoganglionic” gut segments.

Intestinal aganglionosis is caused by molecular defects in extracellular signals and their receptors, intracellular molecular cascades and diverse classes of transcription factors (Heanue and Pachnis, 2007). The receptor tyrosine kinase (RTK) Ret, in association with the GPI-anchored co-receptors GFRα1-4, forms signalling receptor complexes that are activated by the GDNF family of ligands (GFLs) (Airaksinen et al., 1999; Baloh et al., 2000). *Ret*, *Gfr*α*1* and *Gdnf* are critical for the development of the mammalian ENS since deletion of each of these genes results in total intestinal aganglionosis (Schuchardt et al., 1994; Moore et al., 1996; Pichel et al., 1996; Sanchez et al., 1996; Enomoto et al., 1998). Consistent with the role of *Ret* in mouse ENS development, mutations in the human homologue have been identified in approximately 50% of familial cases of HSCR (Amiel et al., 2008; Emison et al., 2010). Although no changes in the coding sequence of *RET* can be identified in the remaining familial cases, reduced expression of this gene is thought to be a contributing factor in most cases of HSCR (Grice et al., 2005; Miao et al., 2010).

In mouse embryos, *Ret* is not expressed during the early stages of vagal NC migration but the gene is induced in pENCCs as they approach the dorsal aorta (Durbec P. L. et al., 1996). During colonisation of the gut by ENCCs *Ret* expression is maintained in undifferentiated progenitors and in enteric neurons, but the gene is down-regulated in glial cells (Hao and Young, 2009; Laranjeira and Pachnis, 2009). Analysis of the phenotype of mutant mice has demonstrated that Ret signalling is necessary for the survival of the early progenitors of the ENS within the foregut, while *in vitro* and organ culture studies have suggested that Ret promotes the proliferation and migration of ENCCs (Taraviras et al., 1999; Young and Newgreen, 2001; Natarajan et al., 2002; Gianino et al., 2003). The role of Ret signalling in the differentiation of enteric neurons and the formation of functional neuronal circuits within the gut has been described by Lasrado et al. (2017). However, the elimination of the majority of early ENS progenitors upon *Ret* deletion has prevented the *in vivo* analysis of the role of this signalling pathway on the proliferation and migration of ENCCs at later stages of embryogenesis. These studies require the generation and analysis of hypomorphic and conditional mutant alleles of *Ret* which can by-pass the early requirement of this gene for the survival of early ENS progenitors (Uesaka et al., 2008; Uesaka and Enomoto, 2010).

*c-Ret* is expressed in two main isoforms *Ret*^9^ and *Ret*^51^, which differ in their C-terminal tail sequence from residue 1062 (Y1062): with *Ret*^9^ containing 9 downstream amino acids versus 51 different amino acids in *Ret^51^.* Both isoforms have been shown to be important in ENS development. In order to provide critical insights into the role of Ret in ENS development we have previously generated the monoisoformic alleles *Ret*^9^ and *Ret*^51^, which express one of the two main Ret isoforms, Ret^9^, Ret^51^, respectively (de Graaff et al., 2001). Using targeted mutagenesis in embryonic stem cells, monoisoformic mouse lines were generated expressing either Ret^51^ or Ret^9^ isoforms. Mice expressing only Ret^9^ isoform were phenotypically normal and displayed a normal ENS. However, mice homozygous for the *Ret*^51^ isoform (i.e., expressing only Ret^51^ and no Ret^9^) lack enteric ganglia from the distal 2/3 of the colon and show intestinal obstruction and megacolon, cardinal features of HSCR (de Graaff et al., 2001). Ret and other signalling pathways such as EDNRB as well as transcription factors such as Sox10 have been shown to be important for ENS development (Barlow et al., 2003; Bondurand et al., 2006). The binding of ligands (Durbec P. et al., 1996; Trupp et al., 1996) activates the Ret receptor and triggers multiple transduction pathways through different adaptor proteins. It has been hypothesised that altered adapter protein binding, to isoform-specific carboxyl-terminal sequences, mediates downstream signalling activation and the developmental functions of each isoform (Wong et al., 2005; Jain et al., 2010). However, the molecular mechanisms underlying the differing functions of the individual isoforms remain unclear. Therefore, to better understand the consequences of *Ret* mutations in ENS development, we have analysed the phenotype of *Ret^51/51^* homozygous animals during embryogenesis and explored the mechanisms by which *Ret* mutations lead to aganglionosis. These experiments suggest that normal *Ret* activity is required *in vivo* for the efficient migration of ENCCs throughout the gastrointestinal tract and for their normal proliferation and neuronal differentiation. Moreover, we demonstrate that enteric neurons in *Ret^51/51^* homozygous mice have defective axonogenesis and thus abnormal neuronal circuits within the gut wall. We also show that the neuronal deficit in the gut of *Ret^51/51^* mutants can be reproduced in clonogenic cultures of multilineage ENS progenitors isolated from embryonic gut and that this deficit can be rescued by expression of the missing Ret^9^ isoform. Based on these findings, we suggest that abnormal intestinal function in HSCR patients is not restricted to the aganglionic hindgut but is likely to involve more proximal “normoganglionic” gut segments. Our data also argue that the neurogenic deficit of multilineage ENS progenitors from HSCR patients is not irreversible and can be restored by correcting the relevant molecular defect.

## Materials and Methods

### Animals

The generation of the *Ret*^51^ (de Graaff et al., 2001) and *Rosa26^stopYFP^* (MGI:2449038) (Srinivas et al., 2001) alleles and the *Tg*Wnt*1*^Cre^** (MGI:2386570) transgene have been described previously (Danielian et al., 1997). Since the *Rosa26* (MGI:104735) and *Ret* (MGI:97902) loci are linked on mouse chromosome 6^1^, and the *Ret*^51^ and *Rosa26^stopYFP^* alleles have been generated independently, we devised a breeding strategy to generate a recombinant chromosome that carries both alleles. More specifically, we first crossed *Ret^+/+^;Rosa26^+/StopYFP^* and *Ret^+/51^;Rosa26^+/+^* animals to generate mice heterozygous for both the *Ret*^51^ and *Rosa26^StopYFP^* alleles. These mice were then crossed to wild-type animals and their progeny were screened for co-transmission of *Ret*^51^ and *R26^StopYFP^*. Approximately 2% (1/50) of these mice were positive for both alleles suggesting that meiotic recombination generated a recombinant chromosome carrying both alleles (YFP and *Ret*^51^ knocked-in alleles). This was subsequently confirmed by further breeding of these animals to wild-type mice and genotyping of their progeny. The day of vaginal plug detection was considered to be E0.5. Mouse studies were carried out under the authority of a UK Home Office Project License in a Home Office designated facility.

### Immunostaining

Immunostaining on whole mount gut preparations was performed as described previously (Bondurand et al., 2006). Briefly, dissected guts were fixed for 2 h in 4% PFA at 4°C, washed in PBT (PBS + 0.1% Triton X100) and incubated in PBT containing 10% heat-inactivated sheep serum (PBT-HISS) at room temperature (RT) for a minimum of 1 hour. The specimens were then incubated overnight (O/N) at 4°C with either rabbit anti-GFP (Molecular Probes; 1:1000) or the mouse anti-b-Tubulin III, TuJ1 (Covance, United Kingdom; 1:1000) diluted in PBT-HISS. After washing, secondary antibody (in PBT-HISS) was applied for 2-3 h at RT or O/N at 4*^o^*C (anti-rabbit Alexa Fluor 488 or anti-mouse Alexa Fluor 568 (both Molecular Probes, 1:1000). After several washes in PBT, samples were photographed using a Leica GFP microscope and documented using OpenLab software.

Fixed and cryoprotected embryos were frozen in OCT compound and sections were cut at 12 μm thickness using a MICROM HM 560 cryostat. For immunolabelling, sections were postfixed with 4%PFA, washed with PBS + 0.1% Triton X100 (PBT), and blocked for 30 min using blocking solution (1%BSA, 0.15% glycine in PBT). All primary antibodies were diluted using the blocking solution and were applied O/N. Secondary antibodies were applied for 1–4 h at RT. Slides were mounted using anti-fade mountant (Vectashield + Dapi; Vector Laboratories).

Short term cultures were fixed for 10 min at RT with 4% PFA, washed with PBT and treated with primary and secondary antibodies as described above.

The antibodies used were as follows: TuJ1 (Covance, United Kingdom 1:1000); GFP (anti-mouse or anti-rabbit Molecular Probes, 1:1000); GFAP (rabbit; DAKO, United States, 1:400); B-FABP (rabbit; kind gift from Thomas Muller, 1:1000); SOX10 (mouse; cell line kindly provided by Dr. David Anderson, 1:1), anti-HuC&D (mouse, Molecular Probes, 1:300); anti-BrdU antibody (rat; Oxford Biotechnology, 1:500), anti-phospho-histone 3 (PH3, rabbit; Chemicon, 1:500). Secondary antibodies used were anti-mouse Alexa Fluor 488 or 568, anti-rabbit Alexa Fluor 488 or 568, anti-rat Alexa Fluor 568 (all Molecular Probes, 1:1000 dilution).

Immunostaining of gut short term cultures or sections from embryos exposed to BrdU were performed as follows. Following incubation with primary and secondary antibodies as above, cell/sections were post-fixed in 4% PFA for 10 min, washed with PBT and then incubated in freshly prepared 2 M HCl in PBT at RT for 15 min. After washing with 1x PBS for 2 × 5 min at RT, cells/sections were incubated with primary and secondary antibodies (rat anti-BrdU followed by anti-rat Alexa Fluor 568). Cells/sections were washed and mounted in Vectashield + Dapi.

### BrdU Incorporation

BrdU (Sigma-Aldrich, Feltham, United Kingdom) stock solution (10 mg/ml made in 0.9% NaCl) was injected intraperitoneally (10 μl/gm) into pregnant mice and embryos were harvested 1 h after the BrdU injection. Embryos were processed for sectioning as described above.

### Short Term Enteric Nervous System Cultures

Short term cultures were performed as described previously (Bondurand et al., 2006). Guts were dissected from E11.5 or E14.5 embryos in L15 medium (PAA Laboratories, Yeovil, United Kingdom), washed 2x in Ca^2+^ and Mg^2+^ free PBS (Phosphate Buffered Saline; Roche) and incubated with dispase/collagenase (Roche, 0.5 mg/ml in PBS) for 3 min at RT (E11.5) or 45 min at 37*^o^*C (E14.5). The tissue was then washed in 1xPBS, dissociated into single cells by pipetting and plated onto fibronectin-coated (20 μg/ml, Sigma) 8-well permanox slide wells (VWR, Leicestershire, United Kingdom) in OptiMEM (Gibco, Invitrogen, Horsham and Loughborough, United Kingdom) supplemented with L-glutamine (1 mM; Gibco Invitrogen, Horsham and Loughborough, United Kingdom) and 1% penicillin/streptomycin (Gibco Invitrogen, Horsham and Loughborough, United Kingdom). These cultures were maintained for 2–3 h in a 37*^o^*C incubator with 5%CO_2_, and then processed for immunostaining.

To establish short term ENS cultures from perinatal animals, guts were incubated in L15 medium on ice for 20–30 min. The outer muscle layers containing myenteric plexus was peeled, rinsed in 1xPBS and digested with 1mg/ml of Collagenase, Type IV (Sigma-Aldrich, Feltham, United Kingdom) for 1 h at 37*^o^*C. The tissue was washed with PBS, resuspended in OptiMEM, supplemented with L-glutamine (1 mM; Gibco Invitrogen, Horsham and Loughborough, United Kingdom) and 1% penicillin/streptomycin (Gibco Invitrogen, Horsham and Loughborough, United Kingdom) and plated onto fibronectin coated dishes and cultured 2–3 h.

### Neuronal Outgrowth

For neuronal outgrowth assays, E11.5 guts from +/*Ret*^51^ x + /*Ret*^51^ heterozygous crosses were dissociated individually as mentioned above and plated sparsely onto dishes coated with 1 mg/ml poly-D-lysine hydrobromide (Sigma-Aldrich, Feltham, United Kingdom) and 100 mg/ml Laminin (Sigma-Aldrich, Feltham, United Kingdom) in Neurobasal medium (Invitrogen, United Kingdom) with N2 and B27 supplements (Invitrogen, United Kingdom), 1% penicillin/streptomycin (Gibco Invitrogen, Horsham and Loughborough, United Kingdom) and 1 mM Glutamine (Gibco Invitrogen, Horsham and Loughborough, United Kingdom). The cultures were maintained for 6 days in an incubator at 37*^o^*C with 5% CO_2_. Cultures were then fixed and processed for immunolabelling with TuJ1 antibody. Neuronal outgrowths were measured using the Neuronal outgrowth programme (Metamorph) on an Axiophot fluorescence microscope.

### DiI Labelling of Postnatal Guts and Estimation of Neurite Length

Guts isolated from postnatal animals from +/*Ret*^51^ x + /*Ret*^51^ heterozygous crosses were fixed in 4% PFA for 20 min at RT. A small crystal of Dil (1,1′-didodecyl 3,3,3′,3′-indocarbocyanine perchlorate, Cell Tracker, Molecular Probes) was placed at 2 different locations on the midgut and the colon. Organs were then placed in 4% PFA for 6 days in a 37*^o^*C incubator, washed with PBS and mounted onto glass slides for photography. The axons which projected furthest at P1, in both the oral and aboral directions from the DiI crystal placed in the midgut, were measured using the Neurite outgrowth programme (Metamorph). 10–12 axons from 6 control (*Ret*^+/+^) and mutant *Ret^51/51^* guts, obtained from 4 independent litters, were measured both orally and aborally in control and mutant genotypes.

### Generation and Analysis of Enteric Nervous System Progenitor Cells From *Ret^51/51^* Guts

Retroviruses expressing *Ret*^9^ or *Ret*^51^ were generated by cloning human cDNA for *Ret*^9^ or *Ret*^51^ into the Not1 site of the pMX-IRES-GFP vector. Isolation of EPCs, analysis of clonal cultures and pMX-IRES-GFP-retroviral transductions have been described previously (Bondurand et al., 2003). Briefly, guts were isolated individually from +/*Ret*^51^ x + /*Ret*^51^ heterozygous crosses, enzymatically dissociated and cultured until neurophere-like bodies formed at approximately day 7. Genotypes were pooled and transduced with the retroviruses for either 7 h (embryonic cells) or O/N (for postnatal cells) and FACS sorted for GFP. GFP + EPCs were plated at “clonal” density (i.e., about 200–300 cells per well of 6 well dish) and cultured for 7–10 days. Colonies were then immunostained and analysed as described above (36). Efficiency of transduction varied with the batch of retrovirus (i.e., control GFP RV vs. Ret^9^ RV vs. Ret^51^ RV). However, with an MOI of 2–3, the efficiency was found to be approximately 5% in embryonic gut cells versus 1% in postnatal gut cells. For all EPCs experiments, 3 independent litters were used. In each litter, cells of the same genotype were pooled. Post FACS, 6–11 colonies with 50–100 cells were counted in each experiment.

### Imaging

All samples were analysed using either an Axiophot epifluorescence microscope (Zeiss), or an Axiovert microscope (Zeiss). Whole mounts guts were analysed using an Olympus/Leica stereoscope. All figures were compiled using Adobe Photoshop software.

### Statistical Analysis

Data are expressed as mean ± standard error of the mean. Statistical analysis was performed using GraphPad Prism software (GraphPad). Intergroup differences were evaluated by Welch’s *t*-test, in addition to ANOVA in Figure 6. In cases where sample sizes were small, we further applied Mann–Whitney *U* tests which validated the results obtained via *t*-test analysis. Statistical differences were considered to be significant if *p*-value was less than 0.05.

**FIGURE 1:**
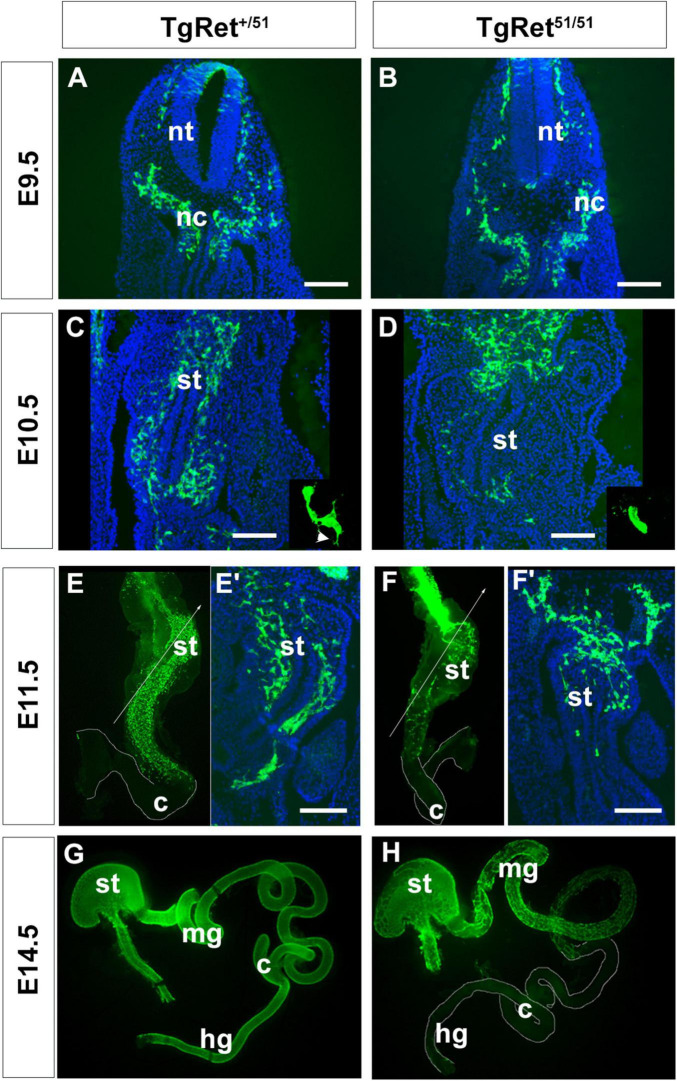
Delayed migration of ENS progenitors along the gut of *Ret*^51/51^ mutant embryos. **(A,B)** Immunostaining of representative cryosections through the foregut of control (Tg*Ret*^+/51^) and Tg*Ret*^51/51^ E9.5 embryos with GFP-specific antibodies. At this stage no difference was detected in the distribution of GFP-labelled vagal neural crest cells (nc) and pENCCs between control and mutant embryos. **(C,D)** GFP immunostaining of sections through the foregut of E10.5 control (Tg*Ret*^+/51^) and Tg*Ret*^51/51^ embryos. In control (Tg*Ret^+/51^)* embryos, ENCCS are distributed throughout the foregut mesenchyme but in Tg*Ret*^51/51^ mutants they fail to migrate much beyond the gastroesophageal junction. Insets show high power images of isolated GFP^+^ cells from control (Tg*Ret^+/51^)* and mutant embryos, respectively. Arrowhead in inset **(C)** shows projections in control ENCC. **(E,F)** Whole mount preparations of gut dissected from control (Tg*Ret*^+/51^) and Tg*Ret*^51/51^ E11.5 embryos, immunostained for GFP showing that migration of Tg*Ret*^51/51^ ENCCs is delayed relative to control Tg*Ret*^+/51^ cells. **(E’,F’)** Sections through the foregut of control Tg*Ret*^+/51^ and Tg*Ret*^51/51^ embryos that correspond to the lines shown respectively in **(E,F)**. **(G,H)** Whole mount preparations of gut dissected from control Tg*Ret*^+/51^ and Tg*Ret*^51/51^ E14.5 embryos immunostained for GFP. Control ENS progenitors have colonized the entire gut but *Ret*^51/51^ progenitors have colonized only the foregut and the proximal half of the small intestine. Embryos from 3 litters per stage were used (approximately 4–6 per genotype). The embryos were isolated, genotyped individually and analyzed separately using immunolabelling. Scale bar: 200 μm. nc, neural crest; nt, neural tube; st, stomach; mg, midgut; c, caecum; hg, hindgut. Tg*Ret*^+/51^ represents cells or embryos from genotype *Wnt1*^cre/+^*;R26R*^stop/YFP^*;Ret^+/51^* and Tg*Ret*^51/51^ represents cells or embryos from genotype *Wnt1*^cre/+^*;R26R*^stop/YFP^*;Ret^51/51^*.

**FIGURE 2:**
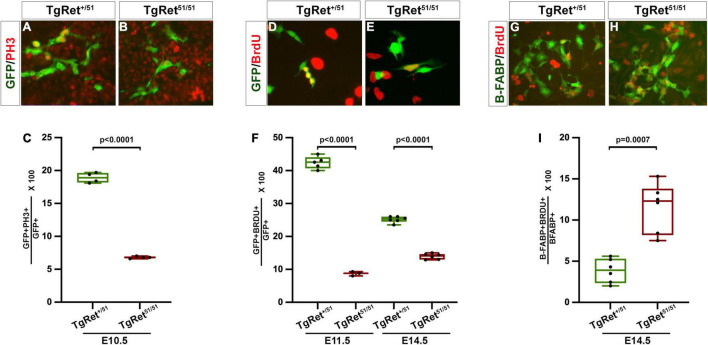
Reduced proliferation of ENCCs homozygous for the *Ret*^51/51^ mutation. **(A,B)** Short term cultures of dissociated gut from control (Tg*Ret*^+/51^) and Tg*Ret*^51/51^ E10.5 embryos immunostained for GFP and PH3. **(C)** Quantification of the percentage of double positive cells (GFP^+^PH3^+^ {green/red}) in the two genotypes *n* = 4. **(D,E)** Short term cultures established from dissociated guts from control (Tg*Ret*^+/51^) and Tg*Ret*^51/51^ embryos that had previously been exposed to BrdU. **(F)** The fraction of GFP^+^BrdU^+^ {green/red} cells was quantified for E11.5 and E14.5 embryos (*n* = 5, 6 resp). **(G,H)** Short term cultures of dissociated gut from control (Tg*Ret*^+/51^) and Tg*Ret*^51/51^ E14.5 embryos immunostained form B-FABP (green) and BrdU (red) *n* = 6. **(I)** Quantification of the percentage of B-FABP^+^BrdU^+^ double immunostained cells. GFP (green), BrdU (red). Statistical analysis performed by Welch’s *t*-test. Tg*Ret*^+/51^ represents cells or embryos from genotype *Wnt1*^cre/+^*;R26R*^stop/YFP^*;Ret^+/51^* and Tg*Ret*^51/51^ represents cells or embryos from genotype *Wnt1*^cre/+^*;R26R*^stop/YFP^*;Ret^51/51^*.

**FIGURE 3:**
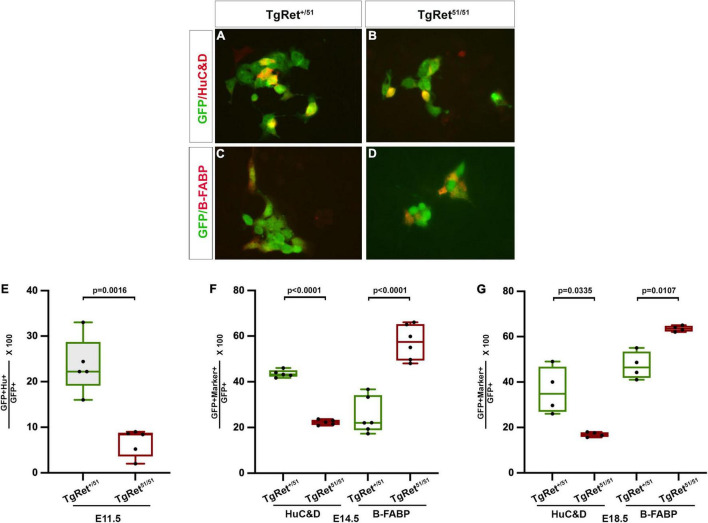
Normal *Ret* activity is required for enteric neuron differentiation. **(A,B)** Short term cultures of dissociated gut from control Tg*Ret*^+/51^ **(A)** and Tg*Ret*^51/51^ **(B)** E11.5 embryos showing the co-expression of GFP (green) with a neuronal marker HuC/D (red). **(C,D)** Short term cultures of gut from control Tg*Ret*^+/51^ **(C)** and Tg*Ret*^51/51^ **(D)** E14.5 embryos showing co-expression of GFP (green) and a glial marker B-FABP (red). **(E–G)** Quantification of the percentage of GFP cells co-expressing either HuC/D or B-FABP at various time points. Graph showing percentage of GFP co-expressing HuC/D at E11.5 **(E)**. A significantly smaller fraction of *Ret*^51/51^ GFP^+^ cells have differentiated into neurons. Quantification of the percentage of GFP cells co-expressing HuC/D or B-FABP in short-term cultures of dissociated gut from control, Tg*Ret*^+/51^ and Tg *Ret*^51/51^ E14.5 embryos **(F)**. Quantification of percentage of GFP cells co-expressing HuC/D or B-FABP from whole mount of myenteric plexus of E18.5 **(G)** from control Tg*Ret*^+/51^ and Tg*Ret*^51/51^ embryos. Although the fraction of *Ret*^51/51^ GFP^+^ cells that have differentiated into neurons is reduced, the fraction expressing glial markers is increased at both E14.5 and E18.5. Embryos from 4 litters were used for analysis. Statistical analysis performed by Welch’s *t*-test. Tg*Ret*^+/51^ represents cells or embryos from genotype *Wnt1*^cre/+^*;R26R*^stop/YFP^*;Ret^+/51^* and Tg*Ret*^51/51^ represents cells or embryos from genotype *Wnt1*^cre/+^*;R26R*^stop/YFP^*;Ret^51/51^*.

**FIGURE 4:**
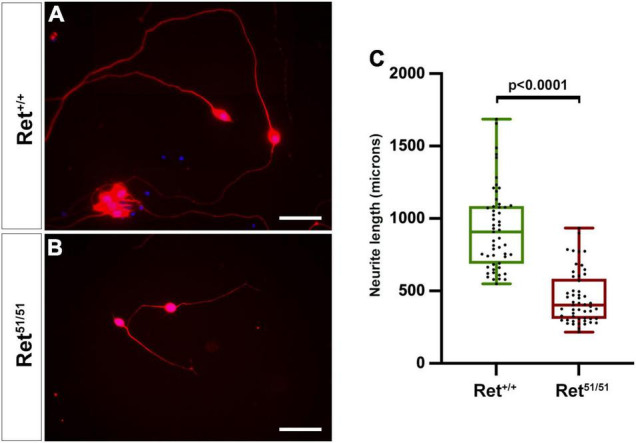
Neuritogenesis is compromised in *Ret*^51/51^ neurons maintained in culture. **(A,B)** Cultures of enteric neurons from gut of control (*Ret*^+/+^) and *Ret*^51/51^ embryos immunostained for TuJ1 (red). **(C)** Graph showing the average neurite length of control (*Ret*^+/+^) and *Ret*^51/51^ neurons from gut cultured for 6 days. *Ret*^51/51^ neurons have significantly shorter processes. Embryos from 3 litters of E11.5 *Ret*^+/51^ heterozygous crosses were analysed. (*n* = 6 for each genotype; 53 axons each). Scale bar: 200 μm. Statistical analysis performed by Welch’s *t*-test.

**FIGURE 5:**
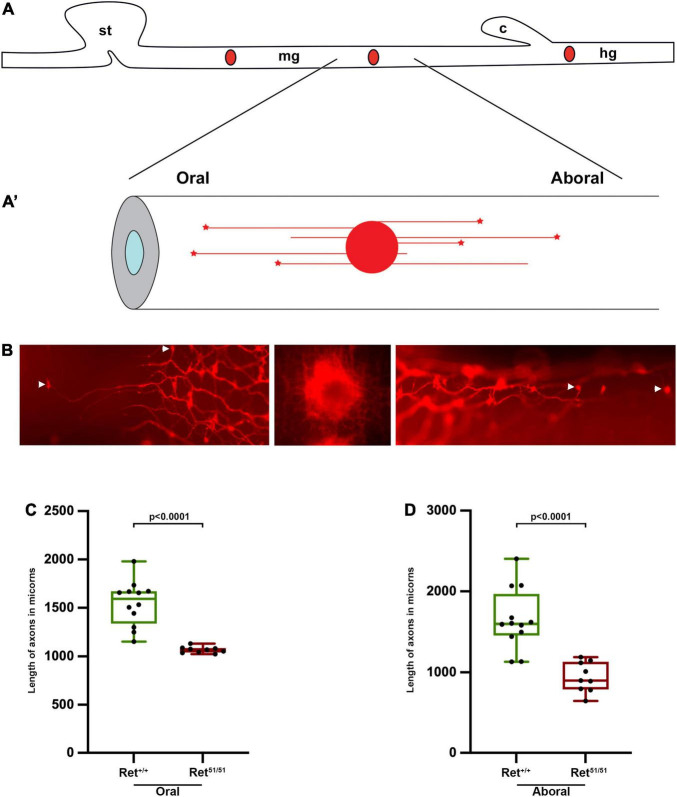
Reduced neurite length of *Ret*^51/51^ enteric neurons **(A,A’)** Schematic representation of DiI labelling and retrograde DiI labelling of individual enteric neurons and their processes. **(B)** Fluorescent images of DiI labelled enteric neurons in the gut of perinatal animals. Middle panel shows the site of DiI crystal, left and right panels show individually labelled oral and aboral enteric neurons respectively. Arrowheads point to the cell body showing length of processes of control (*Ret*^+/+^) and *Ret*^51/51^ orally **(C)** and aborally projecting **(D)** neurons in the gut of perinatal animals. P1 pups (*n* = 6 for each genotype) from 4 litters were used for analysis; 9–12 axons were measured in the oral and aboral directions. Statistical analysis performed by Welch’s *t*-test.

**FIGURE 6:**
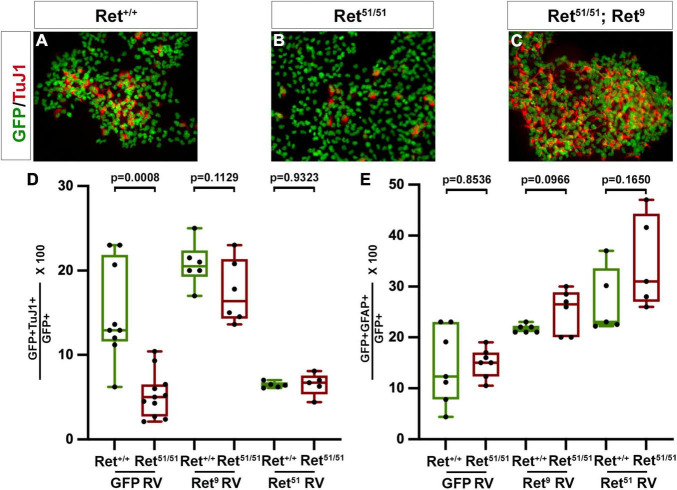
*Ret* activity is critical for neuronal differentiation of multilineage ENS progenitors. **(A,B)** Colonies established from control (*Ret*^+/+^) and *Ret*^51/51^ EPCs were immunostained for GFP (green) and TuJ1 (red). Neuronal differentiation was dramatically reduced in the *Ret*^51/51^ colony. **(C)** Expression of *Ret*^9^ isoform in colonies from *Ret*^51/51^ EPCs rescues their neuronal differentiation deficit. Graph **(D)** shows quantification of neuronal differentiation in control (*Ret*^+/+^) and *Ret*^51/51^ EPC colonies after transduction with control GFP, *Ret*^9^ and *Ret*^51^ retroviruses (GFP RV, *Ret*^9^ RV and *Ret*^51^ RV). **(E)** Quantification of glial differentiation in control (*Ret*^+/+^) and *Ret*^51/51^ EPC colonies after transduction with control GFP, *Ret*^9^ and *Ret*^51^ retroviruses (GFP RV, *Ret*^9^ RV and *Ret*^51^ RV). Neuronal differentiation was rescued on expressing the *Ret*^9^ isoform but this was reduced when *Ret*^51^ isoform was expressed in control (*Ret*^+/+^) EPCs. EPCs were isolated from 3 independent Litters. genotypes were pooled and 6–11 colonies counted post transduction, FACS and culture. Statistical analysis performed by Welch’s *t*-test. ANOVA (and multiple comparison) analysis of equivalent colonies transduced with either control GFP, *Ret*^9^ or *Ret*^51^ retroviruses in shown in Supplementary Table 1.

## Results

### Delayed Colonisation of the Gut by *Ret^51/51^* Homozygous Neural Crest Cells

To explore the mechanisms of aganglionosis in HSCR cases resulting from *RET* mutations we compared the development of the ENS in embryos in which NC cells were either heterozygous or homozygous for the *Ret*^51^ mutation. To facilitate these studies, we lineally marked early NC cells by generating, via breeding, a recombinant chromosome carrying both alleles (*R26*^StopYFP^*Ret^51^*; for breeding strategy see section “Materials and Methods”). Enteric neurogenesis was then compared in *Tg*Wnt*^1–cre^* transgenics that were either heterozygous *R26*^StopYFP^*Ret^+/51^* (hereafter called control; Tg*Ret^+/51^*) or homozygous for the *R26*^StopYFP^*Ret^51/51^* mutant allele (hereafter called Tg*Ret^51/51^*). Embryos from 3 litters per stage were used. The embryos were isolated genotyped individually and analysed separately using immunolabelling. In E9.0-9.5 control Tg*Ret^+/51^* embryos, YFP^+^ vagal NC cells were present lateral to the neural tube and ventrolaterally to the dorsal aorta (Figure 1A). A similar distribution of YFP^+^ cells was observed in equivalent sections from Tg*Ret^51/51^* embryos (Figure 1B), indicating that the pre-enteric stages of vagal NC cell migration remain unaffected by the *Ret*^51^ mutation. In E10.5 control (Tg*Ret^+/51^*) embryos a large number of YFP^+^ cells were found scattered throughout the mesenchyme of the foregut, from the ventral side of the dorsal aorta (Figure 1C top), through the wall of the stomach and up to the duodenum (Figure 1C bottom). In mutant littermates however, YFP^+^ cells were present in the mesenchyme of the gastroesophageal junction (Figure 1D) with very few cells found in more distal foregut regions. Consistent with their migratory behaviour, in control (Tg*Ret^+/51^*) embryos, YFP^+^ cells projected prominent cellular processes into the surrounding mesenchyme but such processes were conspicuously absent from mutant (Tg*Ret^51/51^*) YFP^+^ cells (compare insets in Figures 1C,D and arrowhead in Figure 1C).

As expected, in E11.5 control (Tg*Ret^+/51^*) embryos YFP^+^ cells had colonised the foregut and most of the small intestine with the front of NC cell migration approaching the caecum (Figures 1E,E’). In contrast, noticeably fewer YFP^+^ cells were present in the stomach and the small intestine of mutant littermates and the front of migration was located at more proximal gut regions (Figures 1F,F’). In addition to their reduced number and delayed migration, mutant ENCCs and their progeny were tightly packed and only occupied a small sector of the intestinal radial axis (data not shown).

In control (Tg*Ret^+/51^*) embryos colonisation of the gastrointestinal tract by ENCCs was completed by E14.5 (Figure 1G; Young et al., 1998; Bondurand et al., 2006). However, in similar stage mutant embryos (Tg*Ret^51/51^*), the front of migration of YFP^+^ cells were still within the small intestine (Figure 1H). Eventually, ENCCs colonised the entire small intestine and the proximal colon of Tg*Ret^51/51^* animals but no colonisation of the distal 2/3 of the colon was observed at any stage. Taken together, these data indicate that despite the timely invasion of the foregut mesenchyme by *Ret^51/51^* ENCCs, the subsequent rostrocaudal migration of these cells is severely compromised. Such migratory deficit is evident throughout the period of colonisation of the gut by ENS progenitors and results in failure of arrival of ENCCs in the distal colon.

### Reduced Proliferation of *Ret^51/51^* Homozygous Enteric Neural Crest Cells

The reduction in the number of ENCCs in the gut of Tg*Ret^51/51^* embryos (Figure 1) raised the possibility that normal *Ret* activity is required *in vivo* for the proliferation of ENS precursors. To examine this possibility, appropriate sections from embryos, obtained from 3 litters, that had been exposed to a short pulse of bromodeoxyuridine (BrdU) prior to harvesting (to mark cells in the S-phase) were double immunostained for YFP and BrdU. In addition, untreated embryos, from 3 litters, were also immunostained for YFP and pH3 (to mark cells in mitosis). No significant difference was observed in the fraction of BrdU-labelled YFP^+^ pENCCS present in sections of control (Tg*Ret^+/51^*) and mutant (Tg*Ret^51/51^*) E9.0-9.5 embryos (data not shown). In contrast, the fraction of pH3^+^GFP^+^ cells in the gut of E10.5 mutant embryos (6.8 ± 0.1%; *n* = 4) was significantly reduced relative to control (Tg*Ret^+/51^*) littermates (18.9 ± 0.4%; *p* < 0.0001; *n* = 4) (Figures 2A,B and graph Figure 2C). Consistent with these results, BrdU incorporation was also reduced in ENS progenitors of E10.5 mutant embryos relative to controls.

To examine the potential role of *Ret* in the proliferation of ENS progenitors at later stages of gut colonisation, the guts of individual BrdU-treated embryos were dissociated into a single cell suspension and plated onto fibronectin-coated tissue culture dishes. Two hours after plating these short-term cultures were fixed and double immunostained for YFP and BrdU. In cultures from E11.5 control (Tg*Ret^+/51^*) embryos, 42.4 ± 0.8% of YFP^+^ cells were also positive for BrdU (*n* = 5) but only 8.7 ± 0.4% of *TgRet^51/51^* YFP^+^ cells were double labelled (YFP^+^BrdU^+^; *n* = 3, *p* < 0.0001) (Figures 2D,E and graph Figure 2F). A significant reduction in the fraction of BrdU^+^YFP^+^ cells was also observed in E14.5 Tg*Ret^51/51^* embryos (control, Tg*Ret^+/51^*, 25.1 ± 0.4%; Tg*Ret^51/51^*mutant, 13.9 ± 0.4; *n* = 6 each, *p* < 0.0001) (Figure 2F) although at this stage the difference in the proliferation rate of YFP^+^ cells from the two genotypes was less pronounced, suggesting that the capacity of ENS progenitors to proliferate recovers partially at later embryonic stages. To explore the cellular basis of such recovery, we examined BrdU incorporation specifically in BFABP^+^ cells which appear in the gut of mouse embryos at around E14.5 and represent progenitors of enteric glia. This analysis showed a significant increase in the proliferation of the BFABP-expressing glial progenitors in the gut of E14.5 Tg*Ret^51/51^* embryos relative to their control (Tg*Ret^+/51^*) littermates (11.5 ± 1.2% in Tg*Ret^51/51^* homozygous guts compared to 3.8 ± 0.6% in control (Tg*Ret^+/51^*) guts; *n* = 6 each, *p* = 0.0007; Figures 2G,H and graph Figure 2I). Together, these experiments show that normal *Ret* activity is required to maintain the appropriate levels of proliferation of ENS progenitors during gut colonisation.

### Neuronal Differentiation Is Compromised in the Gut of *Ret^51/51^* Embryos

The gut dysmotility observed in HSCR patients is thought to result mainly from the lack of enteric ganglia in the distal colon (Amiel et al., 2008) but the potential effect of *RET* mutations on the differentiation or connectivity of enteric neurons in more proximal “normoganglionic” gut segments is unknown. To begin addressing this issue, we compared neuronal and glial differentiation in the gut of control Tg*Ret^+/51^* and Tg*Ret^51/51^* embryos. For this, short-term cultures of dissociated gut from individual YFP-expressing embryos were double immunostained for YFP and either pan-neuronal (TuJ1 or HuC/D) or glial (B-FABP or GFAP) markers. At E11.5, 23.6 ± 2.7% of YFP^+^ cells from the gut of control (Tg*Ret^+/51^*) embryos co-expressed HuC/D while only 6.6 ± 1.3% of YFP^+^ cells from mutant (Tg*Ret^51/51^*) littermates were positive for this marker (*p* = 0.0016; *n* = 5 each; Figures 3A,B and graph Figure 3E). Similar results were obtained using the pan-neuronal marker TuJ1 (34.4 ± 0.2% of YFP-expressing cells were positive for TuJ1 in control (Tg*Ret^+/51^*) embryos vs. 19.7 ± 0.31% in Tg*Ret^51/51^* embryos; *p* < 0.0001, *n* = 6, Supplementary Figure 1). Reduced neuronal differentiation was also observed in the gut of E14.5 Tg*Ret^51/51^* embryos (22.2 ± 0.5% of YFP^+^ cells co-expressed HuC/D in the gut of Tg*Ret^51/51^* animals compared to 43.5 ± 0.7% in control Tg*Ret^+/51^* littermates; *p* < 0.0001; *n* = 5 each; Figure 3F). Interestingly at this stage, the percentage of YFP^+^ cells co-expressing the glial marker B-FABP in the gut of Tg*Ret^51/51^* embryos was increased (57.3 ± 3.1%) relative to heterozygous (Tg*Ret^+/51^*) controls (25.1 ± 3.2%; *p* < 0.0001, *n* = 6; Figures 3C,D and graph Figure 3F). Additionally, the percentage of YFP^+^ cells co-expressing GFAP was similar to B-FABP (35.6 ± 1.1% in control Tg*Ret^+/51^* embryos versus 57.9 ± 1.2% in Tg*Ret^51/51^* embryos *p* < 0.0001, *n* = 4; Supplementary Figure 1). Consistent with this observation, the fraction of YFP^+^ cells co-expressing *Sox10* [which marks both undifferentiated progenitors and glial cells (Paratore et al., 2001) was also increased (63.2 ± 1.0% in Tg*Ret^51/51^* animals vs. 38.4 ± 2.4% in controls (Tg*Ret^+/51^*); *p* = 0.0007, Supplementary Figure 1)]. A reduced number of neurons and increased number of glial cells was also observed in whole mount preparations of myenteric plexus from Tg*Ret^51/51^* E18.5 animals [HuC/D: 36.2 ± 5.2% in controls (Tg*Ret^+/51^*) versus 16.9 ± 0.5% in Tg*Ret^51/51^* mutants, *p* = 0.0335 and B-FABP: 47.2 ± 3.0% in controls (Tg*Ret^+/51^*) versus 63.5 ± 0.6% in Tg*Ret^51/51^; p* = 0.0107; *n* = 4; Figure 3G]. Taken together, these studies reveal a specific deficit in neuronal differentiation in the gut of mutant Tg*Ret^51/51^* homozygous animals.

### Axonal Defects of Enteric Neurons in *Ret^51/51^* Embryos

Normal development of neuronal circuitry within the gut wall is critical for the peristaltic and secretomotor activity of the intestine (Sasselli et al., 2013) and, as expected, both functions are severely compromised within aganglionic gut segments of HSCR patients (Furness, 2006). To begin addressing the state of neuronal circuits in more proximal “normoganglionic” gut segments in an HSCR animal model, we initially compared neurite development in cultured enteric neurons from control (*Ret*^+/+^) and *Ret^51/51^* embryos. For this, individually dissociated guts from 3 litters of E11.5 embryos were cultured under conditions that allow robust neurite outgrowth and several days later enteric neurons were fixed and immunolabelled with TuJ1 to highlight their processes. Enteric neurons from control (*Ret*^+/+^) embryos extended neurites that were significantly longer compared to those from *Ret^51/51^* animals [control (*Ret*^+/+^): 930.4 ± 39.1 μm versus *Ret^51/51^* mutant, 451.8 ± 24.8 μm; *p* < 0.0001; *n* = 53 axons from 6 embryos/genotype; Figures 4A,B and graph Figure 4C). These findings suggest that axonogenesis of *Ret^51/51^* homozygous enteric neurons is compromised, raising the possibility that the organisation of neuronal circuits in the ganglionated gut segments of *Ret^51/51^* animals is defective.

To further explore this possibility *in vivo*, we compared neurite length in intact guts from control (*Ret*^+/+^) and *Ret^51/51^* newborn mice. Due to the complexity of the enteric plexus at this stage (Furness, 2006) we were unable to measure the length or follow the trajectory of individual axons using standard immunostaining procedures. To bypass this limitation, we applied the lipophilic dye DiI (1,1′-didodecyl 3,3,3′,3′-indocarbocyanine perchlorate) to specific locations along fixed whole-mount gut preparations with the aim of labelling retrogradely the neurites and the corresponding cell bodies of individual enteric neurons (schematic diagram in Figures 5A,A’; Porter et al., 1997; Sasselli et al., 2013). P1 pups were obtained from 4 independent litters and the axons that extended farthest from a DiI crystal place in the midgut, adjacent to the caecum, were measured. As anticipated, DiI labelled both orally- and aborally-projecting axons which happened to cross the point of DiI application (Figure 5B). Interestingly, in control (*Ret*^+/+^) guts the mean distance of the two furthest away orally or anally projecting neurons was significantly longer relative to their *Ret^51/51^* mutant counterparts (Figures 5C,D). In control (*Ret*^+/+^) animals, orally projecting axons were observed at 1545.8 ± 67.0 mm versus 1067.9 ± 10.9 mm *Ret^51/51^* guts (*p* < 0.0001; *n* = 6 each). Similarly, anally projecting axons were measured at 1649.6 ± 107.7 mm in control (*Ret*^+/+^) gut compared to 941.7 ± 62.1 mm (*p* < 0.0001; *n* = 6 each) in *Ret^51/51^* guts. Taken together with our analysis of enteric neurons in culture, these studies further support the idea that *Ret* activity is required for normal axonogenesis of enteric neurons and the formation of functional neuronal circuits in the mammalian intestine.

### Multilineage Enteric Nervous System Progenitors From *Ret^51/51^* Animals Show Reduced Neuronal Differentiation in Clonogenic Cultures

We and others have previously suggested that multi-lineage progenitors of enteric neurons and glia derived from ganglionated gut segments of HSCR patients could be autotransplanted to the aganglionic colon to restore peristaltic activity (Young et al., 2005; Bondurand et al., 2006). However, our present findings suggest that in addition to the absence of enteric ganglia from the aganglionic gut segments, the ganglionated proximal intestine of HSCR patients is characterised by neuronal deficits, such as a relative reduction in the number of enteric neurons and defective axonogenesis of at least a fraction of enteric neurons. This raises the possibility that upon transplantation into aganglionic gut segments, autologous enteric neural progenitors from “HSCR” patients expressing mutant forms of *Ret* are likely to generate enteric neurons and form ganglia with deficits similar to those detected in the ganglia of origin. To begin addressing this issue we compared neuronal and glial differentiation in clonal cultures of ENS Progenitor Cells (EPCs) isolated from the small intestine of control (*Ret*^+/+^) and *Ret^51/51^* newborn mice (Bondurand et al., 2003). Our previous studies have shown that EPCs can be isolated efficiently from the gut of *Ret^51/51^* mutants (Bondurand et al., 2003) but the extent to which embryonic *Ret^51/51^* EPCs differentiate *in vitro* was not examined in these studies. To address the differentiation capacity of multi-lineage ENS progenitors from *Ret^51/51^* gut, EPCs were isolated from embryonic guts from 3 independent litters from + /*Ret*^51^ x + /*Ret*^51^ heterozygous crosses. From each litter, cells of the same genotype (i.e., *Ret*^+/+^ or *Ret^51/51^*) were pooled and cultured till NLBs were formed (see section “Materials and Methods”). These were transduced using either a control GFP or *Ret*^9^ or *Ret*^51^ containing retrovirus (RV) and after FACS, were allowed to form colonies for 7 days followed by immunostaining with antibodies for pan-neuronal (TuJ1) and glial (GFAP) markers. 6–11 colonies with 50–100 cells were counted and analysed. A significant fraction of cells in control (*Ret*^+/+^) colonies transduced with GFP RV had differentiated into TuJ1^+^ or GFAP^+^ cells (on average 15.1 ± 1.9% neurons; *n* = 9 and 14.4 ± 2.8% glial cells; *n* = 7; Figures 6A,D,E). A similar fraction of glial cells was also present in EPC colonies from *Ret^51/51^* mutant guts (14.8 ± 1.1%; *n* = 7; *p* = 0.8536; Figure 6E). In contrast, the percentage of TuJ1^+^ cells in mutant colonies was significantly reduced (5.31 ± 0.8%, *p* = 0.0008; Figures 6B,D). Moreover, the axonal length of neurons generated from *Ret^51/51^* EPCs was significantly shorter relative to their control (*Ret*^+/+^) counterparts. These findings are consistent with our *in vivo* analysis of *Ret^51/51^* guts and suggest that ENS progenitors from these animals do not differentiate efficiently into neurons.

Similar experiments were conducted using postnatal (P1) guts from control (*Ret*^+/+^) and *Ret^51/51^* embryos. Here, the outer myenteric plexus was isolated as peels, dissociated and cultured until NLBs formed, infected with a GFP containing retrovirus and EPCs plated at clonal densities after FACS. We found that colonies from postnatal EPCs took longer to grow even though the number of colonies formed were not dissimilar between the two genotypes, and as a consequence, differentiation took longer. Therefore, at day 5, differentiation into neurons and glia was minimal in both control (*Ret*^+/+^) and *Ret^51/51^* EPC colonies, therefore analysis was performed at Day 10. However, even at this stage, the neuronal differentiation in *Ret^51/51^* EPCs was reduced compared to control (*Ret*^+/+^) EPCs [6.1 ± 1.2% of EPCs in *Ret^51/51^* colonies versus 12.8 ± 1.5% in control (*Ret*^+/+^) colonies (*n* = 3 each; p = 0.0265; Supplementary Figure 2)]. Expression of earlier markers such as Mash1 and Phox2b however seemed to be similar in both genotypes and appeared at the correct time during differentiation (data not shown). Glial cell differentiation also was not significantly different between the two genotypes. On culturing these colonies for longer periods of time (up to 15 days) more neurons were observed in *Ret^51/51^* EPC colonies but this was still not comparable to equivalent control (*Ret*^+/+^) EPC colonies. Thus far, all our results indicate that the ability of EPCs from *Ret^51/51^* gut are compromised in their ability to form neurons from early embryonic to postnatal stages.

### Expression of the *Ret*^9^ Isoform Rescues the Differentiation Deficit of *Ret^51/51^* Mutant Enteric Nervous System Progenitor Cells

The previous findings argue that ENS progenitors originating from proximal gut segments of HSCR patients and grafted into distal aganglionic gut regions are likely to have reduced neurogenic capacity. A potential means of addressing this limitation would be to genetically modify the grafted cells by expressing a wild-type form of the receptor. To explore the feasibility of this approach, we attempted to correct the genetic deficit of *Ret^51/51^* mutant EPCs by expressing the missing *Ret*^9^ isoform (*Ret^51/51^*;*Ret*^9^) using retroviral transduction (Bondurand et al., 2003) and examined the effect of this genetic modification on neuronal differentiation in clonal cultures. Importantly, the expression of the *Ret*^9^ isoform in *Ret^51/51^* EPCs efficiently rescues the neurogenic deficit of mutant EPCs shown by expression of neuronal marker TuJI (20.8 ± 1.1% in control *Ret*^+/+^ EPCs versus 17.5 ± 1.5% in *Ret^51/51^;Ret^9^* cells, *n* = 6 each, *p* = 0.1129; Figure 6C and graph Figure 6D). Interestingly, no discernible effect on gliogenic differentiation was observed (21.7 ± 0.3% in control *Ret*^+/+^ EPCs versus 25.2 ± 1.8% in *Ret^51/51^;Ret^9^* EPCs, *p* = 0.0966; *n* = 6 each; Figure 6E). These finding suggest that correction of the genetic deficit of EPCs prior to auto-transplantation into aganglionic gut segments is likely to improve their ability to generate appropriate neuronal networks in the gut wall.

Preliminary data from experiments with postnatal EPCs infected with *Ret*^9^ retrovirus indicate that the neuronal differentiation is rescued as in the embryonic EPCs. However, further work needs to be done to analyse their general morphology and neuronal outgrowths.

It was clear from the above experiments that introducing the *Ret*^9^ isoform into *Ret^51/51^* EPCs, rescued the neurogenic potential. We also wanted to investigate the effect of introducing the *Ret*^51^ isoform into control (*Ret*^+/+^) and *Ret^51/51^* EPCs. For this, a retrovirus expressing Ret^51^ was transduced into *Ret^51/51^* EPCs as well as control (*Ret*^+/+^) EPCs and the resulting clonal colonies were analysed as above. There was no significant difference in the percentage of glial cells (27.0 ± 2.9% in control *Ret*^+/+^ versus 34.7 ± 4.1% in *Ret^51/51^;Ret^51^* EPC colonies, *n* = 5 each; *p* = 0.1650, Figure 6E). The same was true for the neurogenic differentiation in *Ret^51/51^* EPCs (6.5 ± 0.6%; *n* = 5) suggesting that introducing the *Ret*^51^ isoform did not rescue the neuronal defect (Supplementary Table 1). However, intriguingly, we found that following introduction of the *Ret*^51^ isoform in control (*Ret*^+/+^) EPCs, in the resulting colonies, neurogenic differentiation was reduced (6.4 ± 0.2%, *n* = 5) similar to the *Ret^51/51^* (*p* = 0.9323) (Figure 6D).

These results support the hypothesis that *Ret*^9^ and *Ret*^51^ represent functionally distinct isoforms which can affect differentiation. The significance of this result is still not clear and more work needs to be done to fully support this finding such as studying the time course of expression, of both isoforms in EPCs, and the effects on differentiation over a longer developmental period.

## Discussion

### Linage Tracing of Neural Crest Cells Shows That Migration Is Delayed in *Ret*^5151^ Embryos

The critical role of the RTK Ret in the development of the mammalian ENS has been well established, and the cellular mechanisms controlled by this signalling pathway *in vivo* are becoming clearer (Lasrado et al., 2017; McCallum et al., 2020). The almost complete elimination of early ENS progenitors in *Ret* null mice (Taraviras et al., 1999) has precluded the analysis of the role of the receptor at later stages of enteric neurogenesis *in vivo* and much of our understanding of its role in ENS development so far has been explored using explant and cell culture assays. However, the role of *Ret* signalling in ENS development using a conditional *Ret* allele inactivated at relatively late stages of embryogenesis has been examined (Uesaka et al., 2007, 2008; Uesaka and Enomoto, 2010). These authors demonstrated that, in addition to promoting survival of early ENCCs, *Ret* signalling is also required for neuronal survival in the colon and suggested that at least some cases of HSCR disease result from a region- and stage-specific elimination of postmigratory and postmitotic enteric neurons. In addition, it was reported that a novel hypomorphic allele of *Ret* results in delayed migration of ENCCs (Uesaka et al., 2008). Notwithstanding these studies, a systematic analysis of the effects of reduced *Ret* signalling throughout enteric neurogenesis is lacking.

Here we have used a genetic lineage tracing system, which marks all NC cells and their derivatives, to analyse enteric neurogenesis in mice in which ENS progenitors are homozygous for *Ret*^51^. Although previous reports had established that *Ret^51/51^* homozygous mice lack enteric ganglia from the distal part of the hindgut (de Graaff et al., 2001), a cardinal feature of HSCR, it was unclear whether the observed colonic aganglionosis in these mice reflected a generalised migratory deficit of ENS progenitors or was due to a hindgut-specific elimination of postmigratory enteric neurons, as suggested previously (Uesaka et al., 2008). Our findings show that pENCCs only expressing *Ret*^51^ were capable of invading the mesenchyme of the proximal foregut but contrary to their Ret-deficient counterparts did not undergo apoptosis. These findings argue that signalling by *Ret*^51^ is sufficient to support survival of ENCCs within the foregut thus bypassing the early apoptotic block in ENS development observed in *Ret* null mice (Taraviras et al., 1999). However, shortly after entering the foregut, ENS progenitors in *Ret^51/51^* mutants showed characteristic deficits in migration, proliferation and differentiation. These studies establish that the cellular outputs of *Ret* signalling during ENS histogenesis are multiple and dose-dependent and reinforce a regulatory role for this receptor in co-ordinating and integrating overlapping cellular processes. Since the initial number of ENCCs that invade the foregut is similar between control and *Ret^51/51^* embryos, we suggest that the ensuing delay in the colonisation of the proximal gastrointestinal tract in the latter is unlikely to result simply from reduced “population pressure” (Landman et al., 2007) but rather suggests a direct role of Ret signalling on ENCC migration. This view is consistent with the disparate morphologies of individual ENCCs, which in control embryos show characteristic migratory features (such as cellular processes that invade the surrounding mesenchyme) that are absent from their mutant counterparts. A potential molecular basis for the population pressure in NC, namely that these cells tend to migrate away from each other upon contact and thus are directed toward less densely populated spaces has been shown by Carmona-Fontaine et al. (2008). Such contact inhibition is likely to be mediated by the Wnt5a planar cell polarity pathway and members of the Rho GTPase family, such as RhoA and Rac1 (Carmona-Fontaine et al., 2008). In a manner perhaps analogous to contact inhibition, explants of embryonic gut already populated by intrinsic ENCCs failed to be colonised by extrinsic NC cells; in contrast, similar stage explants from aganglionic gut were readily colonised by extrinsic NC cells (Hotta et al., 2009). Moreover, *Wnt5a* is known to be expressed at high levels within the intestinal mesenchyme during gut organogenesis (Lickert et al., 2001) while Rac GTPases have a role in Ret signalling and the normal colonisation of the gut by ENS progenitors (Fukuda et al., 2002; Fuchs et al., 2009; Sasselli et al., 2013). Irrespective of the mechanisms, these findings suggest that Ret signalling is required for the repulsive behaviour of ENS progenitors that facilitates the colonisation of the gastrointestinal tract by NC cells during embryogenesis.

### Colonisation of Gut by pENCCS Is Delayed in *Ret^51/51^* Mutants

The apparently normal invasion of the foregut by pENCCS in *Ret^51/51^* homozygous animals is in stark contrast to the profound delay in the colonisation of the rest of the gastrointestinal tube in these mutants, and provides evidence that multiple and genetically distinct mechanisms operate for the colonisation of different gut regions by NC cells. We have previously suggested that GDNF, which during the early stages of gut colonisation is expressed in the mesenchyme of the stomach, ahead of ENCCs, functions as a chemoattractant for a subset of Ret-expressing NC cells that arrive in the dorsal aorta and directs them into the foregut mesenchyme (Young and Newgreen, 2001; Natarajan et al., 2002). It is possible that the complementary expression of RET and GDNF in pENCCs and the stomach, respectively (Natarajan et al., 2002) guarantee the colonisation of the foregut mesenchyme by sufficient numbers of NC cells which, once within the confines of the developing gut, migrate rostrocaudally by directed dispersion as a result of contact inhibition.

Irrespective of the migratory mechanisms that are controlled by the Ret receptor, our data indicate that the colonic aganglionosis of *Ret^51/51^* mutants results from failure of colonisation of the colon by ENS progenitors, as opposed to the elimination of postmigratory cells that have entered the colon. This conclusion is based on two main findings: first, at no point were we able to detect YFP-expressing cells in the distal colon of mutant embryos and, second, we have failed to detect apoptotic cells in the enteric lineages of these mutants. Although it is possible that non-apoptotic mechanisms mediate the death of such cells in distal colon, as suggested previously (Uesaka et al., 2007, 2008) our data suggest that delayed migration of ENCCs is a significant contributing factor for the pathogenesis of at least some cases of HSCR. Moreover, these findings, together with reports by Uesaka et al. (2007, 2008) argue that multiple and complex pathogenetic mechanisms underlie the development of HSCR.

### Ret Signalling Is Important for Neuronal Differentiation

In addition to its role in ENCC migration, our data also provide strong evidence that Ret signalling is required *in vivo* for enteric neuron differentiation. Our suggestion is currently based on the reduced expression of pan-neuronal markers in the gut of *Ret^51/51^* animals. However, the mammalian gut contains a large number of neuronal subtypes that can be distinguished by molecular or functional criteria (Qu et al., 2008; Hao and Young, 2009; Lasrado et al., 2017). Given the widespread expression of *Ret* in undifferentiated ENS progenitors (Durbec P. et al., 1996; Durbec P. L. et al., 1996; Young et al., 1999), it suggests that most, if not all, neuronal subtypes would be underrepresented in the gut of *Ret^51/51^* animals. Reduced neuronal differentiation in the gut of *Ret^51/51^* animals is associated with an increase in the fraction of cells expressing glial markers (Figure 3). This effect could reflect a potential role of Ret signalling in the choice between neuronal and glial cell identities in the mammalian ENS. McCallum et al. (2020) have shown in zebrafish that a high proportion of enteric glia proliferate and can differentiate into neurons. Laranjeira et al. (2011) showed that glial cells dedifferentiated and could give rise to neurons at sites of injury.

Our experiments whereby Ret^51^ was expressed in *Ret^51/51^* EPCs using retroviruses perhaps shed light on this fact. Expressing Ret^51^ in *Ret^51/51^* EPCs with the hypothesis that it could perhaps increase the levels of proteins did not rescue neuronal differentiation. However, intriguingly expressing Ret^51^ in wild-type EPCs caused a drastic reduction in the neuronal differentiation of the wild-type EPCs. This may suggest that Ret^51^ is expressed more in progenitor cells which are glial-like and requires Ret^9^ to initiate neuronal differentiation. Hickey et al. (2009) have shown, using RNA isolation and microarray analyses, that genes were differentially induced in response to *Ret*^9^ and *Ret*^51^ isoforms suggesting that they have different biological roles. Alternative studies have also suggested that these isoforms may perform different functions; Jain et al. (2010) have shown that various tyrosines in *Ret*^9^ and *Ret*^51^ are docking sites for several adaptors and that mutating Y1062 in *Ret^51/51^* caused distal aganglionosis. *RET*^51^ transcripts have also been shown to be increased in human MEN2 tumours suggesting their role in tumour formation (Le Hir et al., 2000). Previous work, where Y1062 was mutated to phenylalanine in monoisoformic *Ret^9/9^* mice, showed that the signalling from Y1062 was a critical regulator for development of ENS and kidneys (Wong et al., 2005). Moreover, replacing Y1062 in a *Ret*^51^ context did not fully rescue the phenotype suggesting that amino acid residues around Y1062, in both isoforms, perhaps determined their function. In these studies, *Ret*^51^ expression delays neuronal differentiation but not glial differentiation. As in the CNS, where neural stem cells are similar to astrocytes and glia-like, perhaps *Ret*^51^-expressing NCC are more stem cell like. *In vivo* studies are limited as the *Ret^51/51^* animals do not survive more than P2 due to the added defects in kidney development as well. As ENS development involves many molecules and factors along with RET, more work is needed to fully understand the specific roles of the isoforms and the effect of other factors on their functions.

### Rescue of Neuronal Differentiation Deficits Maybe Relevant for Cell Based Therapies for Hirschsprung Disease

One of the fundamental issues in the treatment of HSCR disease is the postoperative outcome of the (usually) young patients, which is often poor and characterised by continued severe dysmotility of the gut (Thapar, 2009; vans-Barns et al., 2021). Our analysis of *Ret^51/51^* homozygous mice suggests that defects in the organisation and function of neuronal circuits in proximal “normoganglionic” gut segments could at least partly be responsible for the functional abnormalities of the gut prior, and subsequent, to the surgical resection of the obstructed gut segment. Reduced neuronal numbers, reduced length of neuronal processes seen both in EPCs and in newborn gut are likely to result in reproducible changes in the organisation and function of neuronal circuitry in the gut and provide a potential explanation for the functional abnormalities observed throughout the gut of HSCR patients. Moreover, these observations draw attention to the fact that removal of the aganglionic part of the colon in HSCR patients is not likely to eliminate all potential causes of gut dysmotility and malfunction in postoperative life.

Our current observations are also relevant to efforts to rescue the aganglionic phenotype of HSCR patients or animal models of this condition. We and others have previously suggested that self-renewing multilineage ENS progenitors (such as EPCs) isolated from proximal gut segments of HSCR patients could be expanded *in vitro* and used to colonise distal aganglionic gut segments (Kruger et al., 2002; Bondurand et al., 2003; Young et al., 2005). It is currently unknown to what extent, in such auto-transplantation models, the grafted stem cells would maintain a “memory” of the neuronal deficit present in the segments of origin. Tsai et al. (2011), engrafted prospectively selected enteric neural crest stem cells into a rat model of HSCR disease and found that they differentiated into neurons and glia even though the engraftment was diffuse throughout gut. Lindley et al. (2008) transplanted mouse and human neurospheres into aganglionic hindguts of embryonic mouse and showed that neurons and glia were formed, as were synapses. Human gut mucosal tissue was cultured to form NLBs and transplanted into aganglionic chick and foetal human hindgut *in vitro* to produce ganglia like structures containing enteric neurons and glia (Metzger et al., 2009). These transplantations have been performed using wild-type cells with un-colonised gut serving as aganglionic recipients. Our current data show that progenitors originating from ganglionated gut segment of a HSCR animal model maintain the differentiation deficit in culture, suggesting that ENS stem cells from HSCR patients maintain their genetic characteristics. Importantly, our ability to rescue the differentiation deficit of mutant ENS progenitors, by restoring expression of the missing Ret isoform, establishes the genetic manipulation of ENS stem cells as an approach which, in principle, could restore the ability of these cells to generate efficiently enteric neurons upon transplantation into the gut of HSCR patients.

## Data Availability Statement

The datasets presented in this study can be found in online repositories. The names of the repository/repositories and accession number(s) can be found in the article/Supplementary Material.

## Ethics Statement

The animal study was reviewed and approved by United Kingdom Home Office. Mouse studies were carried out under the authority of a UK Home Office Project License in a Home Office designated facility.

## Author Contributions

DN, NT, and VP designed and planned the experiments. DN and NT conducted the study, collected the data, and along with VP interpreted the data, and drafted the manuscript. CM gave valuable comments, helped in editing manuscript and figures and helped with expression studies. JD helped in analysing data as well as gave advice on statistical analyses. All authors have read and agreed to the version of the manuscript.

## Conflict of Interest

The authors declare that the research was conducted in the absence of any commercial or financial relationships that could be construed as a potential conflict of interest.

## Publisher’s Note

All claims expressed in this article are solely those of the authors and do not necessarily represent those of their affiliated organizations, or those of the publisher, the editors and the reviewers. Any product that may be evaluated in this article, or claim that may be made by its manufacturer, is not guaranteed or endorsed by the publisher.

## Footnotes

1
www.ensembl.org

## Supplementary Material

Supplementary Figure 1 Short term cultures of dissociated guts from control Tg*Ret*^+/51^ and Tg*Ret*^51/51^ embryos were immunostained for different markers. Graph shows the quantification of percentage of GFP cells co-expressing TuJI at E11.5 in Tg*Ret*^+/51^ control (green box) and Tg*Ret*^51/51^ (red box) embryos *n* = 6. This was similar to the neuronal marker HuC/D where a significantly smaller fraction of Tg*Ret*^51/51^ GFP^+^ cells have differentiated into neurons. Quantification of GFP cells co-expressing either GFAP (*n* = 4) or Sox10 (*n* = 4) in E14.5 control Tg*Ret*^+/51^ versus mutant Tg*Ret*^51/51^ embryos also showed that GFAP expression was similar to B-FABP whereby the percentage was higher in mutant versus control. This is also shown with Sox10 expression which labels both undifferentiated cells as well as glial cells. The fraction of Tg*Ret*^51/51^ GFP^+^ cells that have differentiated into neurons is reduced at E11.5 and the fraction expressing glial markers is increased at E14.5. Statistical analysis performed by Welch’s *t*-test. Tg*Ret*^+/51^ represents cells or embryos from genotype *Wnt1*^cre/+^*;R26R*^stop/YFP^*;Ret^+/51^* and Tg*Ret*^51/51^ represents cells or embryos from genotype *Wnt1*^cre/+^*;R26R*^stop/YFP^*;Ret^51/51^*.

Supplementary Figure 2 Reduced neuronal differentiation in *Ret*^51/51^ EPC from postnatal gut myenteric peels. EPCs cultured after transduction of P1 control (*Ret*^+/+^) or *Ret*^51/51^ guts using a GFP retrovirus, and labelled with GFP (green) to mark all infected cells and TuJ1 (red), a pan neuronal marker to label all neurons. **(A)** Colony from control (*Ret*^+/+^) EPC showed normal neuronal differentiation. **(B)** Colony from *Ret*^51/51^ EPC showed reduced neuronal differentiation. **(C)** Quantification of neuronal differentiation in colonies. *n* = 3 for each genotype. Statistical analysis performed by Welch’s *t*-test.
