# Supplementary material for: Multiple Roles of Ret Signalling During Enteric Neurogenesis
Source: Front Mol Neurosci. 2022 May 27;15:832317. doi: 10.3389/fnmol.2022.832317 (PMC9186293; doi:10.3389/fnmol.2022.832317)
Supplement: Supplementary file 1 [file Data_Sheet_1.PDF]

**One way ANOVA (+/+ comparison for TuJ1)**	**$F(2,17)=15.57$**	**$p=0.0001$**
**Tukey's Multiple Comparison**	**+/+_Ret^{9} RV**	**+/+_Ret^{51} RV**
**+/+_GFP RV**	$p=0.0521$	$p=0.0055$
**+/+_Ret^{9} RV**		$p<0.0001$
**One way ANOVA (Ret$^{51/51}$ comparison for TuJ1)**	**$F(2,19)=38.57$**	**$p<0.0001$**
**Tukey's Multiple Comparison**	**Ret$^{51/51}$_Ret^{9} RV**	**Ret$^{51/51}$_Ret^{51} RV**
**Ret$^{51/51}$_GFP RV**	$p<0.0001$	$p=0.7161$
**Ret$^{51/51}$_Ret^{9} RV**		$p<0.0001$

**One way ANOVA (+/+ comparison for GFAP)**	**$F(2,15)=7.144$**	**$p=0.0066$**
**Tukey's Multiple Comparison**	**+/+_Ret^{9} RV**	**+/+_Ret^{51} RV**
**+/+_GFP RV**	$p=0.0933$	$p=0.0055$
**+/+_Ret^{9} RV**		$p=0.3117$
**One way ANOVA (Ret$^{51/51}$ comparison for GFAP)**	**$F(2,15)=18.28$**	**$p<0.0001$**
**Tukey's Multiple Comparison**	**Ret$^{51/51}$_Ret^{9} RV**	**Ret$^{51/51}$_Ret^{51} RV**
**Ret$^{51/51}$_GFP RV**	$p=0.0130$	$p<0.0001$
**Ret$^{51/51}$_Ret^{9} RV**		$p=0.0351$
