# Supplementary figures and images for: Multiple Roles of Ret Signalling During Enteric Neurogenesis
Source: Front Mol Neurosci. 2022 May 27;15:832317. doi: 10.3389/fnmol.2022.832317 (PMC9186293; doi:10.3389/fnmol.2022.832317)

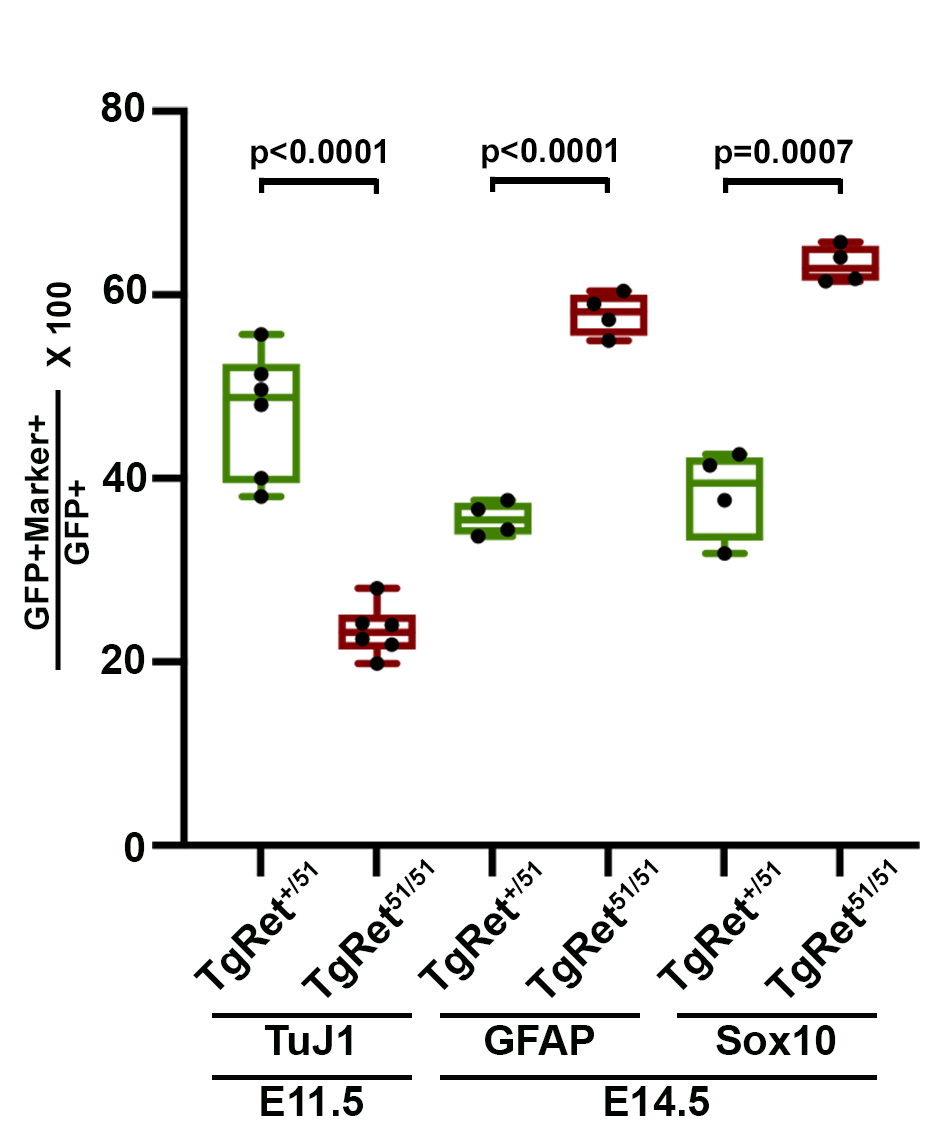

Supplement: Supplementary Figure 1 — Short term cultures of dissociated guts from control TgRet+/51 and TgRet51/51 embryos were immunostained for different markers. Graph shows the quantification of percentage of GFP cells co-expressing TuJI at E11.5 in TgRet+/51 control (green box) and TgRet51/51 (red box) embryos n = 6. This was similar to the neuronal marker HuC/D where a significantly smaller fraction of TgRet51/51 GFP+ cells have differentiated into neurons. Quantification of GFP cells co-expressing either GFAP (n = 4) or Sox10 (n = 4) in E14.5 control TgRet+/51 versus mutant TgRet51/51 embryos also showed that GFAP expression was similar to B-FABP whereby the percentage was higher in mutant versus control. This is also shown with Sox10 expression which labels both undifferentiated cells as well as glial cells. The fraction of TgRet51/51 GFP+ cells that have differentiated into neurons is reduced at E11.5 and the fraction expressing glial markers is increased at E14.5. Statistical analysis performed by Welch’s t-test. TgRet+/51 represents cells or embryos from genotype Wnt1cre/+;R26Rstop/YFP;Ret+/51 and TgRet51/51 represents cells or embryos from genotype Wnt1cre/+;R26Rstop/YFP;Ret51/51. [file Image_1.tif]

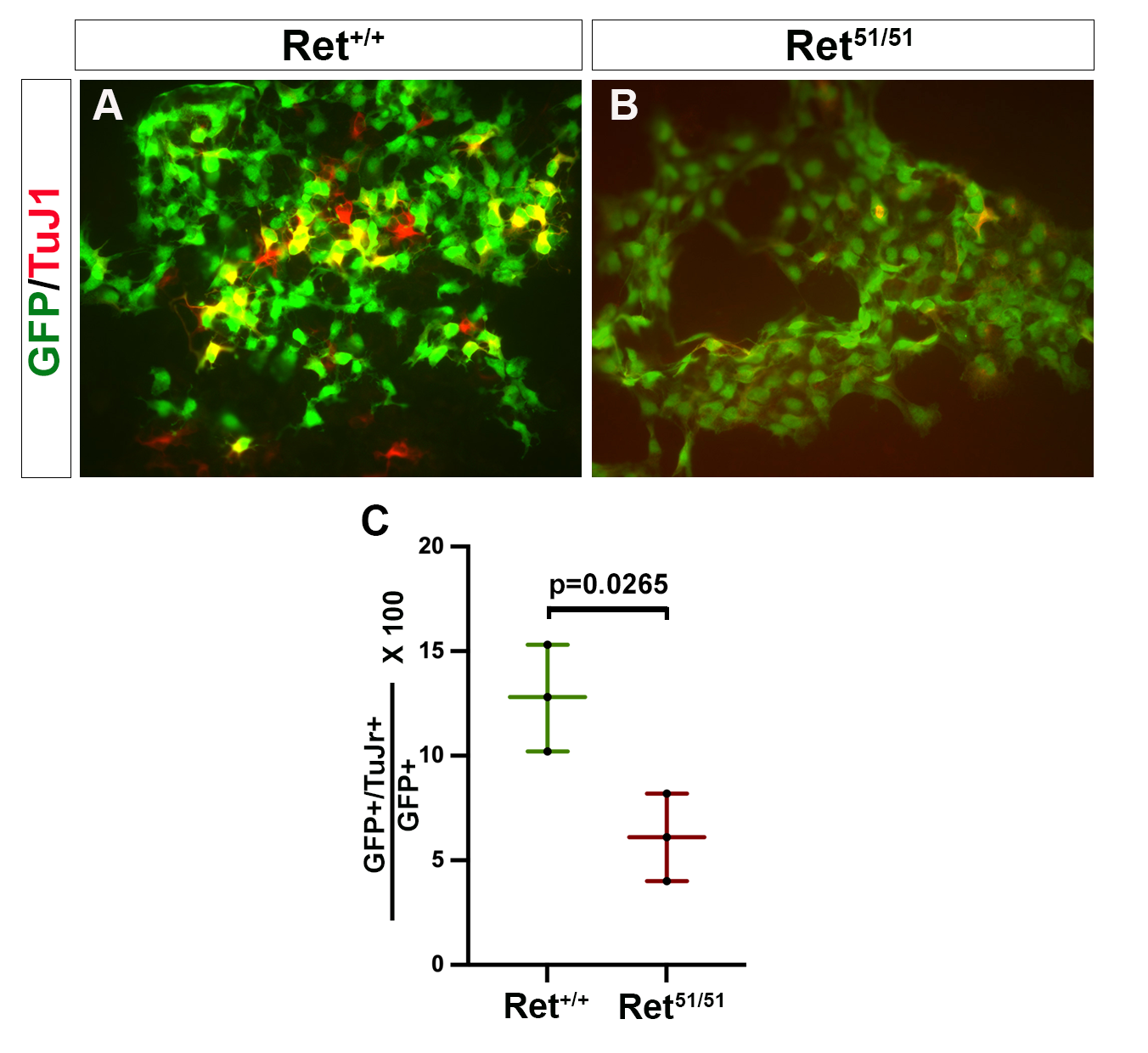

Supplement: Supplementary Figure 2 — Reduced neuronal differentiation in Ret51/51 EPC from postnatal gut myenteric peels. EPCs cultured after transduction of P1 control (Ret+/+) or Ret51/51 guts using a GFP retrovirus, and labelled with GFP (green) to mark all infected cells and TuJ1 (red), a pan neuronal marker to label all neurons. (A) Colony from control (Ret+/+) EPC showed normal neuronal differentiation. (B) Colony from Ret51/51 EPC showed reduced neuronal differentiation. (C) Quantification of neuronal differentiation in colonies. n = 3 for each genotype. Statistical analysis performed by Welch’s t-test. [file Image_2.TIF]
